# Machine Learning and Network Methods for Biology and Medicine

**DOI:** 10.1155/2015/915124

**Published:** 2015-11-29

**Authors:** Lei Chen, Tao Huang, Chuan Lu, Lin Lu, Dandan Li

**Affiliations:** ^1^College of Information Engineering, Shanghai Maritime University, Shanghai 201306, China; ^2^Department of Genetics and Genomics Sciences, Mount Sinai School of Medicine, New York, NY 10029, USA; ^3^Institute of Health Sciences, Shanghai Institutes for Biological Sciences, Chinese Academy of Sciences, Shanghai 200031, China; ^4^Department of Computer Science, Aberystwyth University, Aberystwyth, Ceredigion SY23 3DB, UK; ^5^Department of Radiology, Columbia University Medical Center, New York, NY 10032, USA; ^6^Gastrointestinal Medical Department, China-Japan Union Hospital of Jilin University, Changchun 130033, China

In recent years, many computational methods have been proposed to tackle the problems that arise in analyzing various large-scale high dimensional data in biology and medicine. Useful techniques have been developed by the use of conventional statistical modeling and analysis and have helped to reveal many biological mechanisms. However, with the rapid development of high throughput technologies, biological and medical data generated nowadays are becoming increasingly more heterogeneous and complex. It is therefore necessary to develop more effective and efficient approaches to analyzing such data, requiring more powerful methods like advanced machine learning algorithms and network based methods.

In this special issue, eighteen novel investigations are presented, including a number of newly proposed techniques for up-to-date data analysis and application systems for interesting biological and medical problems.

A computational method was proposed by B. Wang et al. to identify novel candidate genes related to apoptosis. This method first applied shortest path algorithm in a large protein-protein interaction network to search new candidate genes and then the candidate genes were filtered by a permutation test. Twenty-six genes were obtained and analyzed regarding their likelihood of being novel apoptosis-related genes.

F. Yuan et al. proposed a computational method to identify new candidate genes and chemicals based on currently known genes and chemicals related to prostate cancer by applying shortest path approach in a hybrid network which was constructed according to information concerning chemical-chemical interactions, chemical-protein interactions, and protein-protein interactions.

B. Sun et al. designed an analysis pipeline to study the relationships between eight types of damaging protein posttranslational modifications (PTM) and a few human inherited diseases and cancers. The results suggested that some human inherited diseases or cancers might be related to the interactions of damaging PTMs.

Y. Zhan et al. identified a five-gene signature that predicts prognosis in patients with kidney renal clear cell carcinoma (KIRC). The RNA expression data from RNA-sequencing and clinical information of 523 KIRC patients were analyzed. The AUC (area under ROC curve) of the five-gene signature was 0.783 which showed high sensitivity and specificity.

Z. Ji et al. developed a Nonnegative Matrix Factorization (NMF) based feature selection approach (NMFBFS) to identify potential clinical symptoms for HCC patient stratification. The results on 407 HCC patient samples with 57 symptoms showed the effectiveness of the NMFBFS approach in identifying important clinical features, which will be very helpful for HCC diagnosis.

C. Zhang et al. proposed adaptive weight regularized ADSIR for low dose CT reconstruction. Three numerical experiments are carried out for evaluation and comparisons are made with other algorithms.

J. I. Galván-Tejada et al. presented the potential of X-ray based multivariate prognostic models to predict the onset of chronic knee pain. Using X-rays quantitative image-assessments, multivariate models may be used to predict subjects that are at risk of developing knee pain by osteoarthritis.

Y. Cui et al. developed a method called ROC-Boosting to select significant Haar-like features extracted from tongue images for health identification. They analyzed the images of 1,322 tongue cases and selected features focused on the root, top, and side areas of the tongue which can classify the healthy and ill cases.

S. Wang et al. proposed a novel automatic approach for dendritic spine identification in neuron image. The method integrated wavelet based conditional symmetric analysis and regularized morphological shared-weight neural networks. Its good performance and the comparison with existing methods suggest the utility of the method.

S. Yang et al. proposed the use of a combination of edgeR and DESeq to analyze miRNA sequencing data with a large sample size.

R. Hu et al. proposed an automated resource provisioning method, G2LC, for bioinformatics applications in IaaS. It guaranteed applications performance and improved resource utilization. Evaluated on real sequence searching data of BLAST, G2LC saved up to 20.14% of resource.

R. Hu and C. Li proposed an improved PID algorithm based on insulin-on-board estimate using a combinational mathematical model of the dynamics of blood glucose-insulin regulation in the blood system. The simulation results demonstrated that the improved PID algorithm can perform well in different carbohydrate ingestion and different insulin sensitivity situations. Compared with the traditional PID algorithm, the control performance was improved obviously and hypoglycemia can be avoided.

J. G. Rodriguez-Escobedo et al. described the use of the “a priori” algorithm at resolving KIR gene patterns associated with haematological malignancies, previously unrevealed through traditional statistical approaches.

Z. Jiang et al. built a new method to predict chemical toxicities based on ontology information of chemicals. This method was more effective than previous method and provided new insights to study chemical toxicity and other attributes of chemicals.

L. Yuan et al. explored the hidden relationship between miRNAs and imprinted genes in cell pluripotency. They found that the neighbors of imprinted genes on molecular network were enriched in modules such as cancer, cell death and survival, and tumor morphology. The imprinted region may provide a new look for those who are interested in cell pluripotency of hiPSCs and hESCs.

T. Liu et al. reviewed the recent discoveries and advance in the field of evolutional developmental biology in light of the development in large-scale omics studies.

J. A. Vanegas et al. presented a survey on the state-of-the-art text mining approaches to extraction of biomolecular events, which are useful for understanding the underlying biological mechanisms. The popular natural language processing and machine learning methods and tools have been analyzed for this task of phases varied from feature extraction, trigger/edge detection to postprocessing.

Z. Zeng et al. surveyed natural language processing techniques in bioinformatics. First, they searched for knowledge on biology and retrieved references using text mining methods and reconstructed databases. Then, they analyzed the applications of text mining and natural language processing techniques in bioinformatics. Finally, numerous methods and applications are discussed for future use by text mining and natural language processing researchers.

In summary, this special issue collects a number of innovative studies that address various challenging issues in analyzing data in biology and medicine. We hope that this publication will become a landmark in the international development of the relevant literature and also will help encourage more researchers and practitioners to be engaged in this ever increasingly important field.

*Lei Chen* *Lei Chen* *Tao Huang* *Tao Huang* *Chuan Lu* *Chuan Lu* *Lin Lu* *Lin Lu* *Dandan Li* *Dandan Li*

